# Selenium Effect Threshold for Soil Nematodes Under Rice Biofortification

**DOI:** 10.3389/fpls.2022.889459

**Published:** 2022-05-11

**Authors:** Jiaping Song, Xiaodong Liu, Zhangmin Wang, Zezhou Zhang, Qingqing Chen, Zhi-Qing Lin, Linxi Yuan, Xuebin Yin

**Affiliations:** ^1^School of Earth and Space Sciences, University of Science and Technology of China, Hefei, China; ^2^Jiangsu Bio-Engineering Research Center for Selenium/Advanced Lab for Functional Agriculture, Suzhou Institute for Advanced Study, University of Science and Technology of China, Suzhou, China; ^3^Nanjing Institute for FAST/National Innovation Center for Functional Rice, Nanjing, China; ^4^Department of Biological Sciences, Southern Illinois University Edwardsville, Edwardsville, IL, United States; ^5^Department of Health and Environmental Sciences, Xi'an Jiaotong-Liverpool University, Suzhou, China

**Keywords:** selenium, threshold, rice, rhizosphere, biofortification, nematodes

## Abstract

Crop biofortification with inorganic selenium (Se) fertilizer is a feasible strategy to improve the health of residents in Se-deficient areas. For eco-friendly crop Se biofortification, a comprehensive understanding of the effects of Se on crop and soil nematodes is vital. In this study, a rice pot experiment was carried out to test how selenite supply (untreated control (0), 5, 10, 15, 20, 25, 30, 35, 40, 45, 50, 100, or 200 mg Se kg^−1^) in soil affected rice growth, rice Se accumulation, and soil nematode abundance and composition. The results showed that selenite supply (5–200 mg kg^−1^) generally increased the number of rice tillers, rice yield, and Se concentrations in rice grains. In soil under 10 mg kg^−1^ Se treatment, the genus composition of nematodes changed significantly compared with that in the control soil. With increased Se level (> 10 mg kg^−1^), soil nematode abundance decreased significantly. Correlation analysis also demonstrated the positive relationships between soil Se concentrations (total Se and bioavailable Se) with rice plant parameters (number of rice tillers, rice yield, and grain Se concentration) and negative relationships between soil Se concentrations (total Se and bioavailable Se) with soil nematode indexes (nematode abundance and relative abundance of *Tobrilus*). This study provides insight into balancing Se biofortification of rice and soil nematode community protection and suggests the effective concentrations for total Se (1.45 mg kg^−1^) and bioavailable Se (0.21 mg kg^−1^) to soil nematode abundances at 20% level (EC20) as soil Se thresholds. At Se concentrations below these thresholds, rice plant growth and Se accumulation in the grain will still be promoted, but the disturbance of the soil nematodes would be negligible.

## Introduction

Selenium (Se) is an essential element for humans and other animals. It contributes to the protection of liver function and antioxidant defense systems (Brown and Arthur, 2001). Keshan disease and Kashin–Beck disease have been closely linked to low Se intake in humans (Combs, 2001; Fairweather-Tait et al., 2011). Se has been reported to improve symptoms of viral infections, cardiovascular disease, and cancer (Rayman, 2000). The distribution of Se in the soil is uneven. In the Se-rich areas of Ziyang and Enshi in China, the Se concentration in soil reached up to 36.69 and 86.59 mg kg^−1^, respectively (Dinh et al., 2018; Li et al., 2020a). Meanwhile, Se-deficient regions traversing the northeastern region of China until the eastern region of the Tibetan plateau were reported with Se concentrations below 0.20 mg kg^−1^ (Dinh et al., 2018). To obtain enough Se from food, inorganic Se fertilizer has been applied to soil to increase crop Se concentration and hence to overcome the problem of inadequate Se intake by residents in China (Wang et al., 2013a; Wu et al., 2015). However, Se overfertilization is occurring in Se-deficient soils (Winkel et al., 2015; Ros et al., 2016). Excessive Se flux into the soil as a consequence of Se biofortification may exert negative influences on the soil fauna since they remain in the soil for their entire life cycle and are directly affected by the chemicals in the soil (Xu et al., 2022).

Despite the increasing information available on the effects of Se on plants (Lin et al., 2005; Cabral Gouveia et al., 2020), mammals (Benko et al., 2012), and microorganisms (Mojtaba et al., 2015), less is known about the effects of Se on soil fauna. Nematodes are the most abundant metazoans in soil ecosystems, and they are directly involved in the accumulation of organic matter and nutrient cycling (Paz-Ferreiro and Fu, 2016). With more attention to their ecological significance, soil nematodes have been used increasingly as indicators in monitoring soil ecosystem quality (Bongers and Ferris, 1999; Ekschmitt et al., 2001; Neher, 2001). Nematode abundance and composition have been shown to accurately reflect the disturbance caused by fertilizers and heavy metals in soil ecosystems (Bongers et al., 2001; Georgieva et al., 2002; Chen et al., 2003; Zhang et al., 2006; Park et al., 2016).

Limited studies revealed the detrimental effect of Se on soil nematodes. For example, Bakonyi et al. (2003) found that nematode abundance and the number of nematode genera in the experimental group (soil with an ammonium acetate EDTA-extracted Se concentration of 11 mg kg^−1^) were significantly reduced compared with the control group. Se-induced changes in soil nematodes were attributed to omnivorous and predatory nematodes, which could respond quickly to the high-Se stress. In another study (Prins et al., 2019), selenate treatment (80 μM, twice a week) significantly decreased nematode abundance in the rhizosphere soil of the Se-hyperaccumulator plant *Stanleya pinnata*. However, Se was only regarded as a pollutant in the above studies. No study has been conducted yet to reveal the effect of Se on soil nematodes in a soil–plant system under Se biofortification.

Considering the multiple effects of Se (Lv et al., 2021), we hypothesized that soil nematodes will not be disturbed under biofortification with a small amount of Se, while excessive Se may harm soil nematodes and affect plant growth. To test this hypothesis, a rice pot experiment with selenite supplementation at different concentrations was carried out. The objectives were (1) to study the effects of selenite supply on rice plant growth and Se accumulation in the grain, (2) to evaluate the effect of selenite application on soil nematodes, and (3) to determine the soil Se concentration threshold based on nematode response to Se supply.

## Materials and Methods

### Pot Experiment and Plant Growth

A pot experiment using rice was carried out in a greenhouse at the Guangxi Academy of Agricultural Sciences, China, from August to December 2019. The rice cultivar used was *indica* rice Baixiang139, and the rice seedlings germinated and cultured in an incubator under constant temperature (30°C) and light (14 h day^−1^) for 3 weeks were provided by the Guangxi Academy of Agricultural Sciences, China. In 2019, fresh paddy soil was collected from a field in Guigang City, Guangxi. The characteristics of the soil were determined according to the methods of Liu et al. (2018) and are recorded in Table 1. To keep the native nematodes in the soil, fresh soil was used in the pot experiment instead of dry soil. Specifically, the collected fresh soil was broken into small pieces and stirred by a wooden spoon to make it as homogenized as possible. Each pot (diameter: 28.5 cm and height: 27.5 cm) was loaded with 8 kg of fresh weight homogenized soil. Sodium selenite (Na_2_SeO_3_, the major component of Se fertilizer) was used in preparation for a 10 g Se L^−1^ concentrated solution. Then the diluted Se solution (2 L) or ultrapure water (2 L) were added into the pots to attain soil Se concentrations of 0 (untreated control, CK), 5, 10, 15, 20, 25, 30, 35, 40, 45, 50, 100, or 200 mg Se kg^−1^ soil, respectively. The experimental Se concentration range of 0–200 mg kg^−1^ was designed based on the wide soil Se range worldwide, especially the Se-rich hotspots in China, like Enshi in Hubei Province, Ankang in Shaanxi Province, Yichun in Jiangxi Province (Dinh et al., 2018), and overuse of Se fertilizer in Se-deficiency soils (Winkel et al., 2015; Ros et al., 2016). Moreover, the similar wide ranges of Se levels in previous studies were also taken into consideration in this study (Kuperman et al., 2018; Xiao et al., 2018). Three replications were set up at each concentration, with a total of 39 pots. Compound fertilizer (2 g kg^−1^, N:P_2_O_5_:K_2_O ratio of 14:12:14) was added to each pot. After 20 days of aging (Li et al., 2016), three rice seedlings (16 cm, 3 leaves) were planted into each pot. The pots were placed in the greenhouse under natural conditions of illumination and temperature. Se-free water was used for irrigation to simulate flooded paddy conditions. Waterline 4 cm above the soil surface in each pot was set for water content controlling in the whole growth period. After 4 months, mature rice plants were uprooted, and the rhizosphere soil attached to the root surface was collected from each pot carefully (Breidenbach et al., 2016) for later nematode analysis. The rest of potting soil was also collected for Se analysis. The rice plants were washed with deionized water and air-dried. The height of the main culm and the number of tillers were recorded. The grains were separated from plants and dried in an oven at 60°C for 16 h to determine the yield (the dry weight of grains per rice plant).

**Table 1:** Physicochemical properties (dry weight) of the paddy soil used in this study.

**Se** **concentration** **(mg kg^**−1**^)**	**Total** **nitrogen** **(g kg^**−1**^)**	**Total** **phosphorus** **(g kg^**−1**^)**	**Total** **potassium** **(g kg^**−1**^)**	**Hydrolysable** **nitrogen** **(mg kg^**−1**^)**	**Available** **phosphorus** **(mg kg^**−1**^)**	**Available** **potassium** **(mg kg^**−1**^)**	**Organic** **matter** **(g kg^**−1**^)**	**pH**
0.42	1.49	0.92	10.9	60.7	20.3	63.2	21.0	7.05

*The contents of nitrogen, phosphorus, and potassium represent the contents of elements N, P, and K*.

### Soil and Grain Se Analysis

The potting soil was air-dried and homogenized to pass through a 100-mesh sieve. Grains were dehulled, polished, and ground to pass through a 100-mesh sieve. The total Se concentrations in soil or grain were determined using the method described by Long et al. (2020). Briefly, samples (0.2 g soil or grain) were added to a conical flask and then digested for 12 h with 8 ml nitric acid and 2 ml perchloric acid at room temperature. The digested solution was heated on an electric heating plate until white fumes were observed. After cooling, the conical flask walls were rinsed with deionized water, and the solution in the flask was concentrated by reheating until 2 ml solution was left. An aliquot (5 ml) of 12 mol L^−1^ HCl was added to the sample solution to reduce selenate to selenite. Se concentration was determined using the hydride generation atomic fluorescence spectrometry (HG-AFS). The detection limit of this method of Se detection is 0.08 μg kg^−1^. Bioavailable Se in soil was extracted with 0.1 mol L^−1^ KH_2_PO_4_ (Zhao et al., 2005), then the total Se in the supernatant was determined by HG-AFS. National standard reference materials Bush Branch (GBW 07603-GSV-2, Se = 120 ± 20 μg kg^−1^) and Chestnut Soil (GBW 07402-GSS2, Se = 160 ± 40 μg kg^−1^) were used to check the accuracy of Se detection, and the recovery rates ranged from 93.7 to 106.2% in this detection.

### Isolation and Analysis of Soil Nematodes

Nematodes were isolated from the rhizosphere soil samples using the sucrose centrifugal flotation method (Li et al., 2020b). In brief, 100 g soil and 100 ml water were added into a 250-ml centrifuge tube, and a glass rod was used to stir the sample thoroughly. The suspension was centrifuged (810 × *g* for 5 min), and then the supernatant was discarded while the sedimentary soil containing nematodes was retained. An aliquot (100 ml) of sucrose solution (454 g L^−1^) was added to the tube to re-suspend the sediment, and the suspension was centrifuged again (280 × *g* for 5 min). The supernatant containing nematodes was passed through two 500-mesh sieves, then the nematodes retained in the sieves were collected, counted, and identified to the genus level (Bongers, 1988), using an inverted compound optical microscope. Based on trophic type, nematodes were divided into five trophic taxa: bacterivores, algivores, fungivores, herbivores, and omnivores–predators (Yeates et al., 1993). Based on life strategy, nematodes were assigned to five taxa with colonizer–persister (c-p) values ranging from 1 to 5 (Ferris et al., 2001).

### Statistical Analysis

All data represent the means ± standard deviations (SD) of three replicates for each treatment. One-way ANOVA analysis with Tukey multiple range tests for *post hoc* mean comparisons was carried out to identify significant differences (*p* < 0.05) among the different Se treatments. Pearson's correlation analysis was performed to verify the correlations between soil Se concentrations (total Se and bioavailable Se) with indexes of rice plants and soil nematodes. Parametric non-linear regression analysis was used to quantify the relationships between soil Se concentrations (total Se and bioavailable Se) with nematode abundances. The no observed effect concentration (NOEC) was identified as the highest Se concentration showing a response not significantly compared with CK, and the effective concentration at the level of 20% (EC20) was identified as the Se concentration producing a 20% decrease in the measured parameter compared with CK (Kuperman et al., 2018). SPSS 25.0 (IBM, Armonk, NY, USA) was used for statistical analysis, and Origin 2021b (OriginLab, Northampton, MA, USA) was used for visualization.

## Results

### Total and Bioavailable Se Concentration in Soil

The total Se concentrations and bioavailable Se concentrations of potting soils are shown in Table 2. The total Se concentrations were close to nominal Se concentrations, and the bioavailable Se concentrations increased with the elevated total Se concentrations.

**Table 2:** Total and bioavailable Se concentration (dry weight) in the soil after plant harvest.

**Nominal Se** **concentration** **(mg kg^**−1**^)**	**Total Se** **concentration** **(mg kg^**−1**^)**	**Bioavailable Se** **concentration** **(mg kg^**−1**^)**
0 (CK)	0.42 ± 0.06	0.04 ± 0.01
5	7.90 ± 0.51	1.33 ± 0.08
10	9.25 ± 2.35	1.88 ± 0.37
15	14.33 ± 1.78	1.85 ± 0.35
20	21.08 ± 1.31	2.62 ± 0.36
25	25.80 ± 9.52	2.90 ± 0.52
30	27.77 ± 2.54	2.42 ± 0.38
35	30.72 ± 3.35	3.14 ± 0.44
40	32.50 ± 6.83	2.61 ± 0.56
45	46.47 ± 3.48	3.05 ± 0.80
50	57.80 ± 6.21	4.13 ± 0.43
100	135.74 ± 11.99	4.70 ± 0.94
200	196.29 ± 4.38	6.37 ± 0.75

### Rice Plant Growth and Se Accumulation

#### Plant Growth

**Figure 1:**
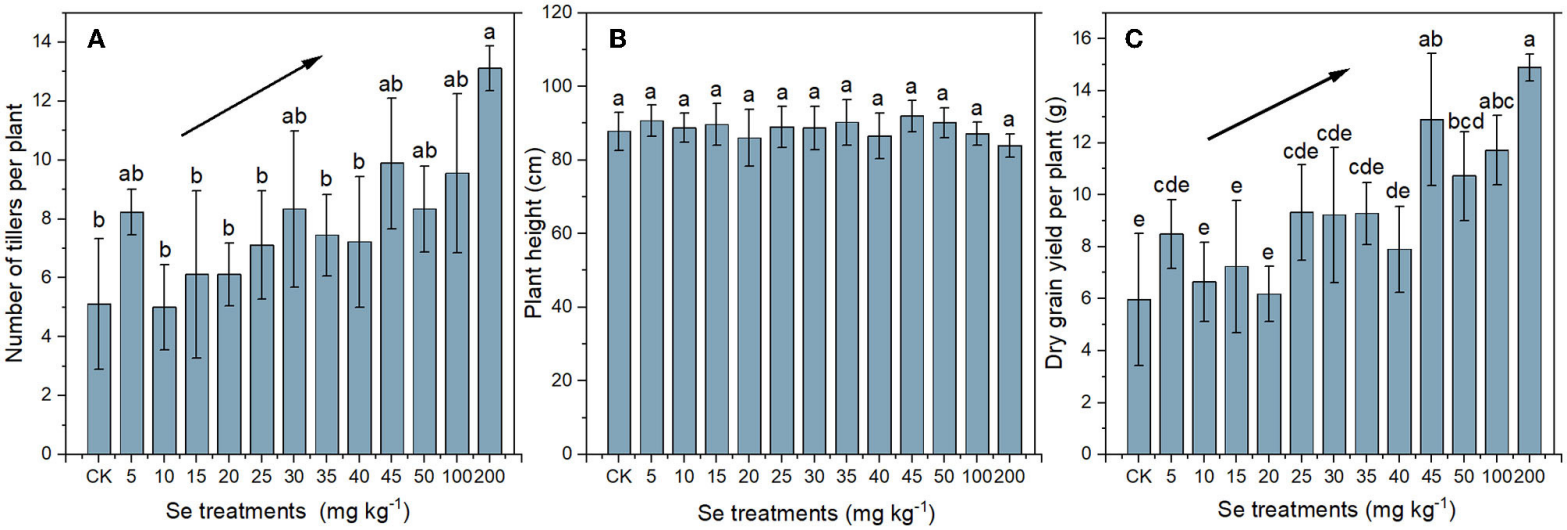
Number of tillers per plant **(A)**, height of the main culm **(B)**, and yield per plant **(C)** of rice plants under different Se treatments. The black lines with arrows indicate increasing trends. Values represent mean ± SD. Any two samples within a bar chart sharing a common letter are not significantly different at the *p* < 0.05 level.

The number of tillers, height of the main culm, and grain yield per plant of rice under different Se treatments are shown in Figure 1. Compared with the CK, no significant difference in tillering was found in rice under low Se treatments ( ≤ 100 mg kg^−1^). However, the tillers number increased to 13.11 ± 0.78 under 200 mg kg^−1^ Se treatment, which was 2.57 times as much as that of CK (5.11 ± 0.22) (Figure 1A). The mean height of the mature rice plants ranged from 83.89 to 91.89 cm, and there was no significant difference in height of plants under different Se treatments (Figure 1B). Variation in grain yield in relation to Se treatment shared a similar trend with tillering variation, and the maximum yield (14.89 ± 0.52 g plant^−1^) was exhibited in rice under 200 mg kg^−1^ Se treatment, being 2.50 times that of the CK yield (5.96 ± 2.54 g plant^−1^) (Figure 1C). In general, the number of tillers and rice yield increased with the increase of supplied Se, indicating that the selenite supply promoted rice growth.

#### Se Concentration in Rice Grain

**Figure 2:**
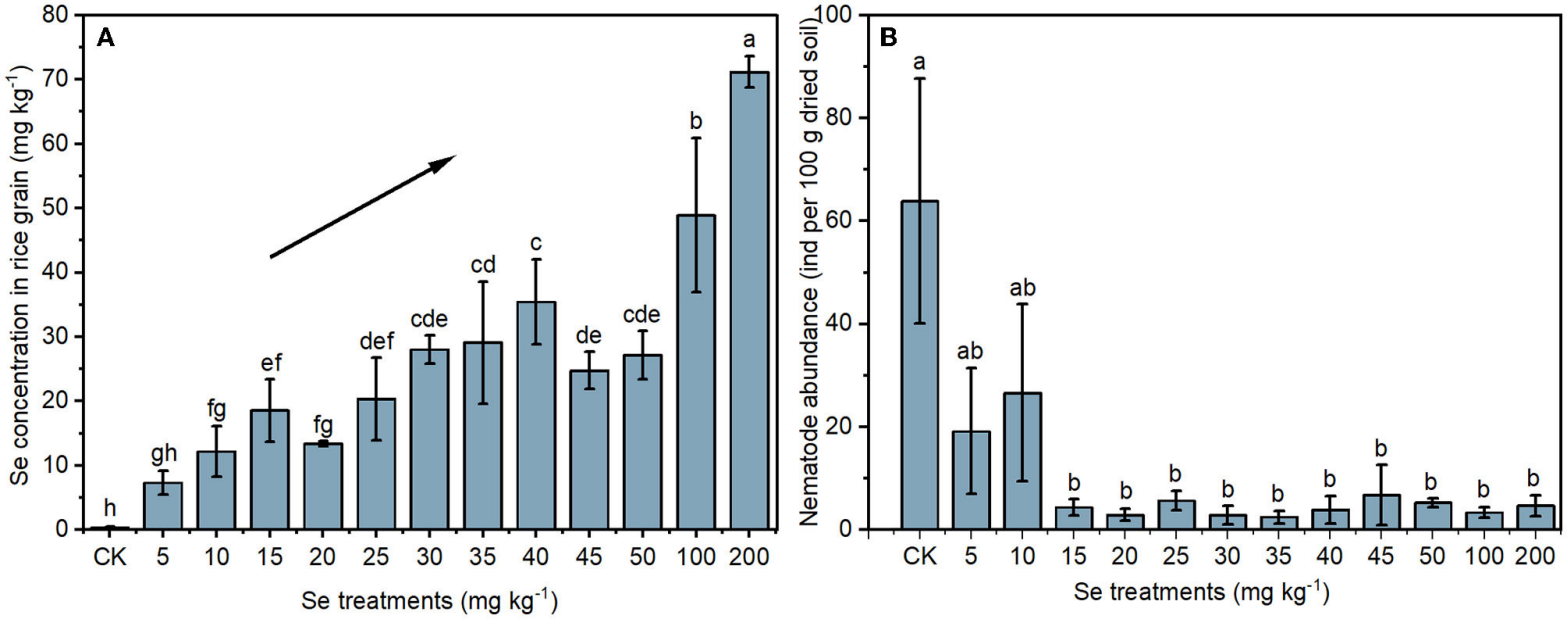
Selenium concentrations in rice grains **(A)** and the abundance of nematodes in soil **(B)** under different Se treatments. The black line with an arrow indicates an increasing trend. Values represent mean ± SD. Any two samples within a bar chart sharing a common letter are not significantly different at the *p* < 0.05 level.

The Se concentrations in rice grains under different Se treatments are shown in Figure 2A. Without Se supply, the Se concentration in CK rice grain was 0.31 ± 0.22 mg kg^−1^. Se concentrations in rice grains increased after Se supply, and the highest concentration of 71.17 ± 2.43 mg kg^−1^ was detected in rice with a 200 mg kg^−1^ Se supply. In general, rice grain Se concentrations increased with the increase of supplied Se.

### Soil Nematode Abundance and Composition

#### Nematode Abundance

**Figure 3:**
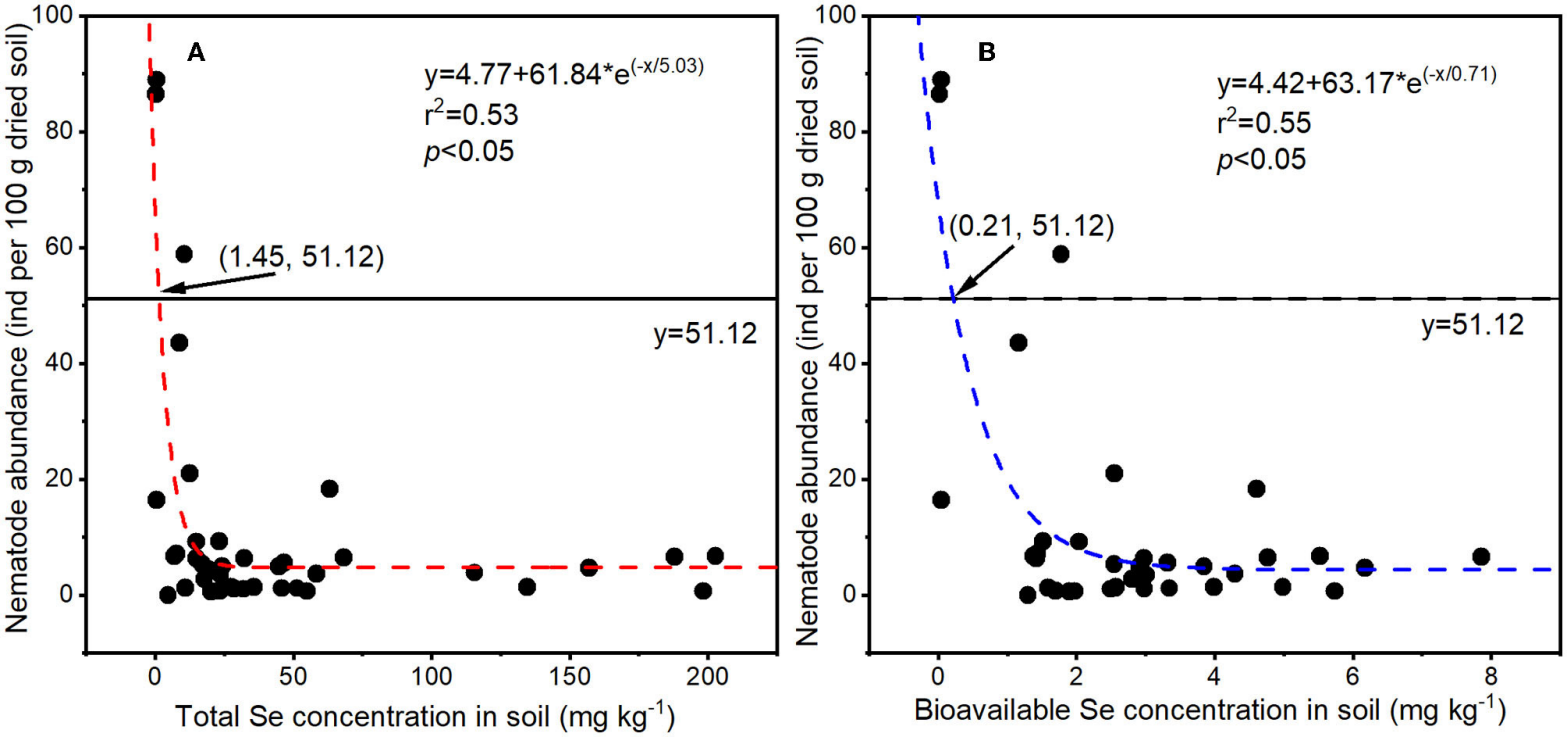
Relationships between soil Se concentrations [total Se **(A)** and bioavailable Se **(B)**] with nematode abundances (*n* = 39). The straight *y* = 51.12 = 63.90 ×80%, 63.90 was the nematode abundance in CK soil, 51.12 was the nematode abundance corresponding to EC20, and 1.45 and 0.21 were EC20 for total Se and bioavailable Se in soil nematode abundance.

To determine the effect of selenite on soil nematodes, the nematode abundance and composition were analyzed. As shown in Figure 2B, soil nematode abundance (number of nematodes in 100 g of dried rhizosphere soil) in the CK group was 63.90 ± 23.79. The nematode abundance in rhizosphere soil under 5 or 10 mg kg^−1^ Se treatment was lower than that in the CK, though not significantly. However, a significant reduction in nematode abundance was observed in soil under higher Se treatments (> 10 mg kg^−1^). The NOEC for total Se and bioavailable Se to soil nematode abundance were therefore 9.25 and 1.88 mg kg^−1^ (actual soil Se level under 10 mg kg^−1^ Se treatment), respectively. Parametric non-linear regression analysis was used to quantify the relationships between actual soil Se concentration (total Se and bioavailable Se) with nematode abundance (Figure 3). The EC20 for total Se and bioavailable Se to nematode abundance were 1.45 and 0.21 mg kg^−1^, respectively.

#### Genus Composition of the Soil Nematodes

**Figure 4:**
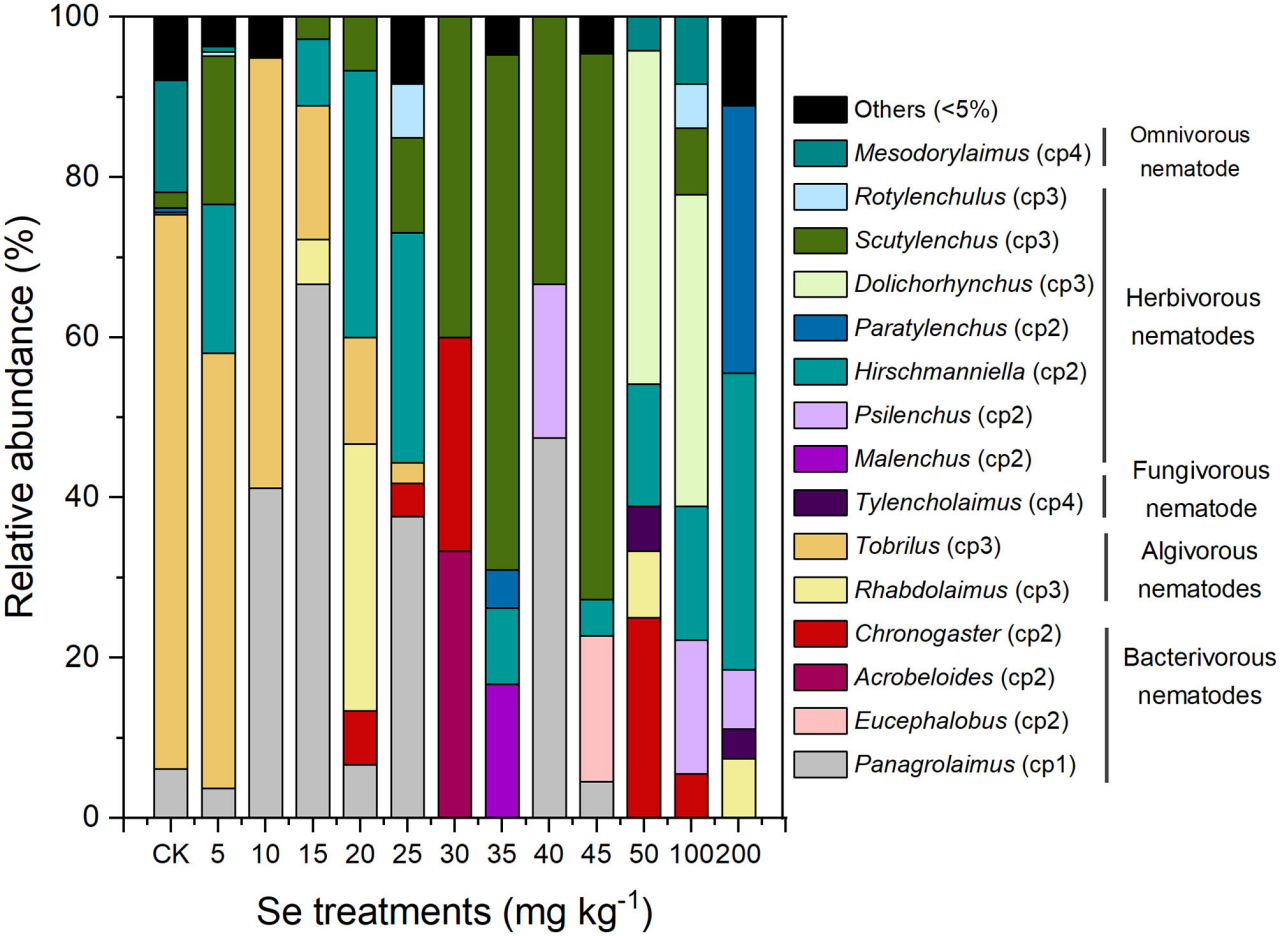
Genus composition of the nematodes in soil under different Se treatments.

A total of 30 nematode genera were detected in all samples, with 15 genera being quite common (relative abundance ≥ 5%). The relative abundance of individual nematode genera is presented in Figure 4, with the rarer 15 genera being classified into one group (others). The genus composition of the soil nematode community varied in soil under different Se treatments. With 5 mg kg^−1^ Se supplement, the soil nematode community exhibited a genus distribution similar to that of the CK in which the predominant genus was *Tobrilus*. The NOEC for soil total Se and bioavailable Se to genus composition of nematodes were therefore 7.90 and 1.33 mg kg^−1^ (actual soil Se level under 5 mg kg^−1^ Se treatment), respectively. Under higher Se treatments (10, 15, or 20 mg kg^−1^), the genus distribution of the nematodes changed, and the predominant genera became *Panagrolaimus* and *Rhabdolaimus*. As the Se concentration increased furtherly (>20 mg kg^−1^), a smaller proportion of algivores and a greater proportion of herbivores were observed, with *Scutylenchus, Dolichorhynchus*, and *Hirschmanniella* being the predominant genera. Overall, excess selenite supplementation shifted the composition of the nematode community from an algivore-dominated one to an herbivore-dominated community and exerted stress on *Tobrilus*.

#### Taxa Composition of the Soil Nematodes

The relative abundance of soil nematode taxa, classified by trophic type or cp-value, is shown in Table 3. With respect to trophic type, the bacterivores, algivores, and herbivores were the dominant taxa in all groups, whereas the fungivores and omnivores–predators were less frequent. In the CK soil, the relative abundances of algivores and herbivores were 69.17 ± 10.42 and 4.39 ± 4.45%, respectively. However, under 200 mg kg^−1^ Se treatment, the relative abundances of algivores and herbivores were 7.41 ± 12.83 and 81.48 ± 32.08%, respectively. Confirming the findings from the genus composition, nematode community composition shifted toward an herbivore-dominated community as the supplied Se concentration increased. With respect to cp-value taxa, the cp-3 nematodes were the predominant taxa in all treatment groups. There was no significant difference in relative abundances of the cp-3 taxon in different treatment groups.

**Table 3:** The relative abundance (%) of taxon in the nematode community.

**Se treatments**	**Trophic taxa**					**cp-value taxa**			
**(mg kg^**−1**^)**	**Bacterivores**	**Algivores**	**Fungivores**	**Herbivores**	**Omnivores–predators**	**cp-1**	**cp-2**	**cp-3**	**cp-4**
0 (CK)	11.49 ± 2.17^a^	69.17 ± 10.42^a^	0.34 ± 0.58^a^	4.39 ± 4.45^a^	14.62 ± 8.03^a^	6.15 ± 5.75^b^	5.61 ± 3.42^a^	73.62 ± 6.06^a^	14.62 ± 8.03^a^
5	3.70 ± 6.42^a^	54.34 ± 42.64^ab^	0.00 ± 0.00^a^	37.64 ± 38.16^a^	4.31 ± 5.96^a^	3.70 ± 6.42^b^	0.00 ± 0.00^a^	91.99 ± 5.37^a^	4.31 ± 5.96^a^
10	42.59 ± 13.09^a^	53.70 ± 18.33^ab^	0.00 ± 0.00^a^	3.70 ± 5.24^a^	0.00 ± 0.00^a^	41.2 ± 15.06^ab^	0.00 ± 0.00^a^	58.80 ± 15.06^a^	0.00 ± 0.00^a^
15	66.67 ± 16.67^a^	22.22 ± 25.46^ab^	0.00 ± 0.00^a^	11.11 ± 19.25^a^	0.00 ± 0.00^a^	66.67 ± 16.67^a^	0.00 ± 0.00^a^	33.33 ± 16.67^a^	0.00 ± 0.00^a^
20	13.33 ± 11.55^a^	46.67 ± 50.33^ab^	0.00 ± 0.00^a^	40.00 ± 40.00^a^	0.00 ± 0.00^a^	6.67 ± 11.55^b^	6.67 ± 11.55^a^	86.67 ± 11.55^a^	0.00 ± 0.00^a^
25	45.94 ± 20.83^a^	2.56 ± 4.44^b^	0.00 ± 0.00^a^	51.50 ± 21.34^a^	0.00 ± 0.00^a^	37.61 ± 34.15^ab^	8.33 ± 14.43^a^	54.06 ± 20.83^a^	0.00 ± 0.00^a^
30	60.0 ± 52.92^a^	0.00 ± 0.00^b^	0.00 ± 0.00^a^	40.00 ± 52.92^a^	0.00 ± 0.00^a^	0.00 ± 0.00^b^	60.00 ± 52.92^a^	40.00 ± 52.92^a^	0.00 ± 0.00^a^
35	0.00 ± 0.00^a^	0.00 ± 0.00^b^	0.00 ± 0.00^a^	100.00 ± 0.00^a^	0.00 ± 0.00^a^	0.00 ± 0.00^b^	21.43 ± 25.75^a^	78.57 ± 25.75^a^	0.00 ± 0.00^a^
40	47.44 ± 46.21^a^	0.00 ± 0.00^b^	0.00 ± 0.00^a^	52.56 ± 46.21^a^	0.00 ± 0.00^a^	47.44 ± 46.21^ab^	19.23 ± 26.92^a^	33.33 ± 57.74^a^	0.00 ± 0.00^a^
45	22.73 ± 32.14^a^	0.00 ± 0.00^b^	4.55 ± 6.43^a^	72.73 ± 38.57^a^	0.00 ± 0.00^a^	4.55 ± 6.43^b^	22.73 ± 32.14^a^	72.73 ± 38.57^a^	0.00 ± 0.00^a^
50	25.00 ± 43.30^a^	8.33 ± 7.22^ab^	5.56 ± 9.62^a^	56.94 ± 38.71^a^	4.17 ± 7.22^a^	0.00 ± 0.00^b^	25.00 ± 43.3^a^	65.28 ± 34.94^a^	9.72 ± 8.67^a^
100	5.56 ± 9.62^a^	0.00 ± 0.00^b^	0.00 ± 0.00^a^	86.11 ± 12.73^a^	8.33 ± 14.43^a^	0.00 ± 0.00^b^	22.22 ± 25.46^a^	69.44 ± 17.35^a^	8.33 ± 14.43^a^
200	0.00 ± 0.00^a^	7.41 ± 12.83^ab^	7.41 ± 12.83^a^	81.48 ± 32.08^a^	3.70 ± 6.42^a^	0.00 ± 0.00^b^	44.44 ± 48.43^a^	48.15 ± 44.91^a^	7.41 ± 12.83^a^

*Different letters indicate a significant difference in the same column at the p <0.05 level. Values represent mean ± SD*.

## Discussion

### The Effects of Selenite on Rice Plant Growth and Se Accumulation

**Figure 5:**
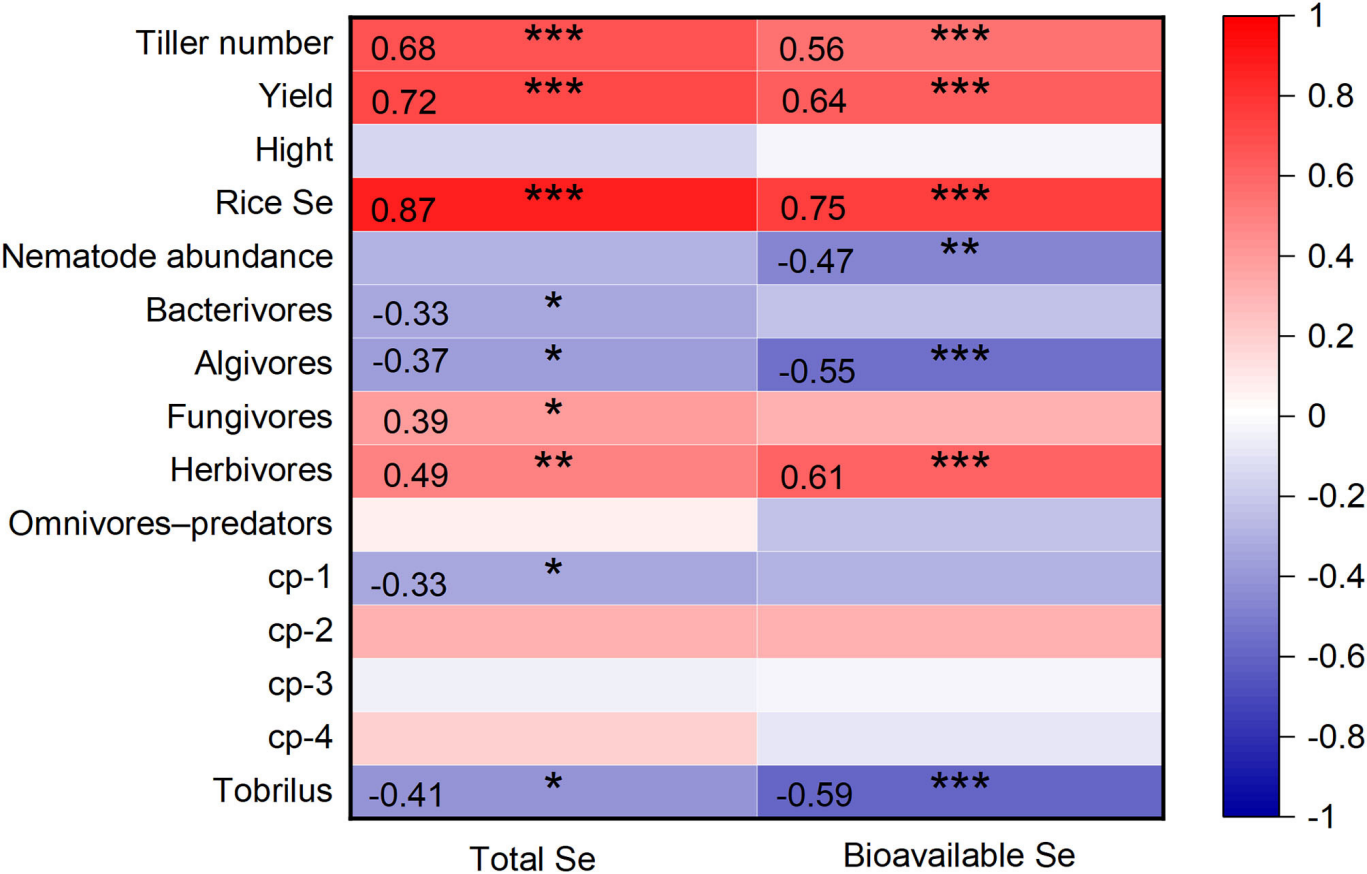
Pearson correlations between the soil Se concentrations (total Se and bioavailable Se) with indexes of rice plant and soil nematodes (*n* = 39). *, **, and *** indicate significant relationships at *p* < 0.05, *p* < 0.01, and *p* < 0.001 level, respectively.

In this study, the number of tillers and rice yield showed a strong positive correlation with soil Se concentrations, including total Se and bioavailable Se (*p* < 0.001, Figure 5). Selenite clearly promoted rice tillering and increased grain yield. Similarly, previous studies have reported that Se supplementation promotes rice growth (Moulick et al., 2016, 2018; Guan et al., 2018). Wang et al. (2013b) reported that rice treated with 21 g Se ha^−1^ produced more tillers per plant, more grains per panicle, bigger grains, and higher yields. The tillering capacity of rice depends mainly on genetic variation and environmental factors (light, temperature, and nutrients). The mineral nutrient Se is beneficial for the formation of rice tillers (Mu et al., 2021). Tiller number controls the panicle number of rice and plays a major role in determining grain yield. Additionally, as a beneficial element for plants, Se is believed to improve the agronomic traits of plants by regulating the activity of photosynthesis and enzymatic antioxidants in plant defense (Feng et al., 2015; Duan et al., 2019).

Selenium is applied to soil worldwide as a feasible and cost-effective method to produce Se-rich crops (Mora et al., 2015). In Finland, the government encourages the use of inorganic Se fertilizer to improve crop nutrition (Alfthan et al., 2015). In China, Se-rich rice was produced by an accurate Se supply (Wu et al., 2015). In a previous study (Dai et al., 2019), Se concentrations of brown rice increased with the elevated soil Se concentration (0.5–20 mg kg^−1^). Similarly, compared with CK, Se treatment (5–200 mg kg^−1^) significantly increased the grain Se concentrations in this study (Figure 2A). Soil total Se and bioavailable Se showed positive correlations with rice grain Se concentration (Figure 5). These correlations might be meaningful for accurate rice Se fertilization, rice grain Se biofortification, and the management of Se-rich soil. It is noteworthy that the rice growth and grain Se accumulation were still promoted by the 200 mg kg^−1^ Se treatment, indicating the high tolerance and accumulation ability of Se by the rice cultivar Baixiang139. Even without Se supply (soil Se concentration of 0.42 ± 0.06 mg kg^−1^), the Se concentration in rice grain reached 0.31 ± 0.22 mg kg^−1^ and beyond the Se-rich rice standard ranging from 0.04 to 0.30 mg kg^−1^ in China (Rich selenium paddy, GB/T 22499-2008). Therefore, the cultivar Baixiang139 could be used to produce Se-rich rice in the future, especially in Se-deficient areas.

### The Effects of Selenite on Soil Nematode

Despite the occurrence of some harmful soil nematodes, overall, nematodes involved in nutrient cycling and energy flow contribute positively to ecosystem processes (Gebremikael, 2016). High nematode abundance has been demonstrated to be a symptom of a healthy soil ecosystem with the general presumption that “the more the better” (Yeates, 2003). The decrease of nematode abundance induced by selenite (Figure 2B) and a negative correlation between nematode abundance and soil bioavailable Se (*p* < 0.01) (Figure 5) were observed. Similarly, high concentrations of trace elements in agricultural soils, such as As, Zn, Cu, and Ni, have been reported to decrease nematode abundance in previous studies (Korthals et al., 1996; Park et al., 2011). The decreased nematode abundance may be achieved through two mechanisms. On the one hand, excessive Se disturbs protein expression and the antioxidant defense system directly in nematodes (Lv et al., 2021). On the other hand, it is likely that the changes in biotic (microorganisms and plant roots) and abiotic (soil properties) factors induced by Se decreased nematode abundance indirectly (Liu et al., 2016).

Besides nematode abundance, the nematode composition is also a focus of this study. A negative correlation between soil Se (total Se and bioavailable Se) with the relative abundance of algivores and bacterivores, and positive correlations between soil Se with the relative abundance of fungivores and herbivores were found (Figure 5). Algivirous nematodes are common in paddy soil (Okada et al., 2011). In this study, the main algivorous nematode, *Tobrilus*, showed a highly sensitive response to Se (Figures 4, 5). Zhao and Neher (2013) conducted a methodical multivariate analysis and then pointed out that nematode genera (*Discolaimium, Discolaimus, Eudorylaimus*, etc.) correlated negatively with the soil Se shows potential in reflecting Se disturbance. Therefore, the sensitive *Tobrilus* can also be used in monitoring environmental Se disturbance in future studies. Herbivores feeding on plant root tissues or root exudates directly or indirectly affect the formation of nodules and mycorrhizae in plants and subsequently downregulate nitrogen fixation and other related functions. According to our results, the rise in herbivorous nematodes may be attributed to increased plant growth induced by Se supplementation. The increase of bacterivores and decrease of fungivores in the soil nematode community may inhibit soil mineralization compared with that in CK since they play a key role in nitrogen mineralization (Ferris et al., 1998). Considering the vital role nematodes play in soil health (Paz-Ferreiro and Fu, 2016), both the changes in abundance and composition of soil nematodes after a high Se supply indicate decreased soil biodiversity and function.

### Balance Between Soil Se Biofortification of Rice With Soil Nematode Community Protection

On account of the low utilization of applied Se by crops, excessive Se might accumulate in soil and do harm to nearby ecosystems (Winkel et al., 2015). The soil fauna, for example, earthworms (Xiao et al., 2018) and collembola (Kuperman et al., 2018), have been reported to be reduced in the soil after Se exposure. The negative effects of excessive Se supply on soil nematodes were also proved by this study. To balance Se biofortification of rice with soil nematode community protection, a soil Se concentration threshold based on nematode response to Se supply is proposed here.

Based on the effect of Se on nematode abundance, the NOEC for soil total Se and bioavailable Se to nematodes was 9.25 and 1.88 mg kg^−1^, respectively (Figure 2B). Based on the effect of Se on nematode genus composition, the NOEC for soil total Se and bioavailable Se was 7.90 and 1.33 mg kg^−1^, respectively (Figure 4). Somogyi et al. (2006) collected soil samples from sunflower fields exposed to artificial selenite pollution for 7 years and analyzed the nematode community. The results demonstrated that the NOEC for soil total Se and bioavailable Se to nematode indexes (abundance, richness, etc.) is 7.25 and 2.09 mg kg^−1^, which is consistent with the findings in this study. Additionally, the EC20 for total Se and bioavailable Se to nematode abundance were 1.45 and 0.21 mg kg^−1^, respectively (Figure 3). Therefore, the lower values (total Se: 1.45 mg kg^−1^ and bioavailable Se: 0.21 mg kg^−1^) were proposed to be soil Se thresholds to keep the nematode from Se disturbance according to the determining of ecological soil screening levels (US Environmental Protection Agency, 2005). At concentrations below soil Se thresholds, plant growth and Se accumulation in the grain will still be promoted, but the disturbance of the nematode community will be negligible. Therefore, the soil Se background concentration should be determined, and the amount of applied fertilizer should be strictly controlled to ensure a low soil Se level after Se fertilization.

Additionally, the method of Se biofortification with inorganic fertilizer can be replaced by approaches that are more friendly to nematodes, like organic Se fertilizers (Se-rich straw and animal manures) (Sharma et al., 2011; Bhatia et al., 2014). Organic fertilizers have been shown to promote soil nematode abundance in studies conducted in grassland and tillage fields (Benkovic-Lacic et al., 2013; Ikoyi et al., 2020). Foliar Se application could be used rather than soil Se application with greater efficiency of Se accumulation in maize (Wang et al., 2013a), wheat (Ros et al., 2016), and soybean (Yang et al., 2003). With ecological safety and economic feasibility (Yang et al., 2021), microbial fortification is also considered to be a nematode-friendly method to produce Se-rich crops.

## Conclusion

Overall, this study demonstrated that soil selenite supply (5–200 mg kg^−1^) promoted plant growth and grain Se accumulation. However, the genus composition of nematodes changed significantly when 10 mg kg^−1^ Se or more were supplied. The abundance of nematodes decreased significantly when 15 mg kg^−1^ Se or more were supplied. These results indicate a potential risk of Se biofortification on soil nematodes. To balance Se biofortification of rice with soil nematode community protection, we suggest that the soil total Se concentration and bioavailable Se concentration after fertilization should be kept below 1.45 and 0.21 mg kg^−1^, respectively. The effects of Se on nematode communities in different agricultural soils growing different crops should be analyzed in future investigations, together with the effects of added Se on soil physicochemical properties.

## Data Availability Statement

The original contributions presented in the study are included in the article/Supplementary Material, further inquiries can be directed to the corresponding authors.

## Author Contributions

XY and LY designed the framework of this study. JS performed the experiments and wrote the manuscript. XY, LY, XL, ZW, ZZ, ZQL, and QC revised this manuscript.

## Funding

This research was supported by the Guangxi Major Special Project of Science and Technique (Guike AA17202026-6), 2019 open project Study on Demonstration Classification Standard of Selenium-Rich Soil and High Selenium Rock of the Key Laboratory of Selenium-Rich Products Development and Quality Control, Ministry of Agriculture and Rural Affairs (Se-2019A02), the Research on the Safe Range of Dietary Selenium Intake (International Cooperation) (D20180031), and the Special Fund for Functional Agricultural Development of Nanjing National Agricultural Innovation Park (NJGJNCY-FAST01).

## Conflict of Interest

The authors declare that the research was conducted in the absence of any commercial or financial relationships that could be construed as a potential conflict of interest.

## Publisher's Note

All claims expressed in this article are solely those of the authors and do not necessarily represent those of their affiliated organizations, or those of the publisher, the editors and the reviewers. Any product that may be evaluated in this article, or claim that may be made by its manufacturer, is not guaranteed or endorsed by the publisher.

## References

[B1] Alfthan G. Eurola M. Ekholm P. Venäläinen E. Root T. Korkalainen K.. (2015). Effects of nationwide addition of selenium to fertilizers on foods, and animal and human health in Finland: from deficiency to optimal selenium status of the population. J. Trace Elements Med. Biol. 31, 142–147. 10.1016/j.jtemb.2014.04.009 24908353

[B2] Bakonyi G. Nagy P. Kadar I. (2003). Long-term effects of heavy metals and microelements on nematode assemblage. Toxicol. Lett. 140, 391–401. 10.1016/S0378-4274(03)00035-3 12676487

[B3] Benko I. Nagy G. Tanczos B. Ungvari E. Sztrik A. Eszenyi P.. (2012). Subacute toxicity of nano-selenium compared to other selenium species in mice. Environ. Toxicol. Chem. 31, 2812–2820. 10.1002/etc.1995 22927138

[B4] Benkovic-Lacic T. Brmez M. Ivezic M. Raspudic E. Pribetić D. Loncaric Z.. (2013). Influence of organic and inorganic fertilizers on nematode communities in cornfield. Bulgarian J. Agricult. Sci. 19, 235–240.

[B5] Bhatia P. Prakash R. Prakash N. T. (2014). Enhanced antioxidant properties as a function of selenium uptake by edible mushrooms cultivated on selenium-accumulated waste post-harvest wheat and paddy residues. Int. J. Recycl. Organic Waste Agricul. 3, 127–132. 10.1007/s40093-014-0074-y

[B6] Bongers T (1988). The Nematodes of the Netherlands. Utrecht: Stichting Uitgeverij Koninklijke Nederlandse Natuurhistorische Vereniging.

[B7] Bongers T. Ferris H. (1999). Nematode community structure as a bioindicator in environmental monitoring. Trends Ecol. Evolution. 14, 224–228. 10.1016/S0169-5347(98)01583-3 10354624

[B8] Bongers T. Ilieva-Makulec K. Ekschmitt K. (2001). Acute sensitivity of nematode taxa to CuSO_4_ and relationships with feeding-type and life-history classification. Environ. Toxicol. Chem. 20, 1511–1516. 10.1002/etc.5620200714 11434292

[B9] Breidenbach B. Pump J. Dumont M. G. (2016). Microbial community structure in the rhizosphere of rice plants. Front. Microbiol. 6:1537. 10.3389/fmicb.2015.01537 26793175 PMC4710755

[B10] Brown K. M. Arthur J. R. (2001). Selenium, selenoproteins and human health: a review. Public Health Nutr. 4, 593–599. 10.1079/PHN2001143 11683552

[B11] Cabral Gouveia G. C. Galindo F. S. Lanza Dantas Bereta M. G. Caroline da Rocha Silva A. Pereira de Brito Mateus M. Souza da Silva M.. (2020). Selenium toxicity stress-induced phenotypical, biochemical and physiological responses in rice plants: characterization of symptoms and plant metabolic adjustment. Ecotoxicol. Environ. Safety. 202:110916. 10.1016/j.ecoenv.2020.110916 32800251

[B12] Chen L. Li Q. Liang W. (2003). Effect of agrochemicals on nematode community structure in a soybean field. Bull. Environ. Contamination Toxicol. 71, 755–760. 10.1007/s00128-003-0196-9 14672128

[B13] Combs G. F (2001). Selenium in global food systems. Br. J. Nutr. 85, 517–547. 10.1079/BJN2000280 11348568

[B14] Dai Z. H. Imtiaz M. Rizwan M. Yuan Y. Huang H. L. Tu S. X. (2019). Dynamics of Selenium uptake, speciation, and antioxidant response in rice at different panicle initiation stages. Sci. Total Environ. 691, 837–834. 10.1016/j.scitotenv.2019.07.186 31326806

[B15] Dinh Q. T. Cui Z. W. Huang J. Tran T. A. T. Wang D. Yang W. X.. (2018). Selenium distribution in the Chinese environment and its relationship with human health: a review. Environ. Int. 112, 294–309. 10.1016/j.envint.2017.12.035 29438838

[B16] Duan M. Y. Cheng S. R. Lu R. H. Lai R. F. Tang X. R. (2019). Effect of foliar sodium selenate on leaf senescence of fragrant rice in South China. Appl. Ecol. Environ. Res. 17, 3343–3351. 10.15666/aeer/1702_33433351

[B17] Ekschmitt K. Bakonyi G. Bongers M. Bongers T. Bostrom S. Dogan H.. (2001). Nematode community structure as indicator of soil functioning in European grassland soils. Eur. J. Soil Biol. 37, 263–268. 10.1016/S1164-5563(01)01095-0

[B18] Fairweather-Tait S. J. Bao Y. P. Broadley M. R. Collings R. Ford D. Hesketh J. E.. (2011). Selenium in human health and disease. Antioxidant Redox Signal. 14, 1337–1383. 10.1089/ars.2010.3275 20812787

[B19] Feng T. Chen S. S. Gao D. Q. Liu G. Q. Bai H. X. Li A.. (2015). Selenium improves photosynthesis and protects photosystem II in pear (*Pyrus bretschneideri*), grape (*Vitis vinifera*), and peach (*Prunus persica*). Photosynthetica 53, 609–612. 10.1007/s11099-015-0118-1

[B20] Ferris H. Bongers T. de Goede R. G. M. (2001). A framework for soil food web diagnostics: extension of the nematode faunal analysis concept. Appl. Soil Ecol. 18, 13–29. 10.1016/S0929-1393(01)00152-4

[B21] Ferris H. Venette R. C. van der Meulen H. R. Lau S. S. (1998). Nitrogen mineralization by bacterial-feeding nematodes: verification and measurement. Plant Soil. 203, 159–171. 10.1023/A:1004318318307

[B22] Gebremikael M. T (2016). Nematodes enhance plant growth and nutrient uptake under C and N-rich conditions. Sci. Rep. 6:32862. 10.1038/srep32862 27605154 PMC5015107

[B23] Georgieva S. S. McGrath S. P. Hooper D. J. Chambers B. S. (2002). Nematode communities under stress: the long-term effects of heavy metals in soil treated with sewage sludge. Appl. Soil Ecol. 20, 27–42. 10.1016/S0929-1393(02)00005-7

[B24] Guan W. W. Dai Q. G. Zhang H. C. Yin X. B. (2018). Effect of selenium fertilization on rice growth and accumulation of heavy metals in rice (*Oryza sativa*). *Soils*. 50, 1165–1169. 10.13758/j.cnki.tr.2018.06.01732144709

[B25] Ikoyi I. Egeter B. Chaves C. Ahmed M. Fowler A. Schmalenberger A. (2020). Responses of soil microbiota and nematodes to application of organic and inorganic fertilizers in grassland columns. Biol. Fertility Soils 56, 647–662. 10.1007/s00374-020-01440-5

[B26] Korthals G. W. Ende A. Megen H. V. Lexmond T. M. Kammenga J. E. Bongers T. (1996). Short-term effects of cadmium, copper, nickel and zinc on nematodes from different feeding and life-history strategy groups. Appl. Soil Ecol. 4, 107–117. 10.1016/0929-1393(96)00113-8

[B27] Kuperman R. G. Checkai R. T. Simini M. Phillips C. T. Higashi R. M. Fan T. W.-M.. (2018). Selenium toxicity to survival and reproduction of Collembola and Enchytraeids in a sandy loam soil. Environ. Toxicol. Chem. 37, 864–853. 10.1002/etc.4017 29078251

[B28] Li J. Peng Q. Liang D. L. Liang S. J. Chen J. Sun H.. (2016). Effects of aging on the fraction distribution and bioavailability of selenium in three different soils. Chemosphere 144, 2351–2359, 10.1016/j.chemosphere.2015.11.011 26606190

[B29] Li M. L. Yang B. Y. Xu K. Y. Zheng D. S. Tian J. C. (2020a). Distribution of Se in the rocks, soil, water and crops in Enshi County, China. Appl. Geochem. 122:104707. 10.1016/j.apgeochem.2020.104707

[B30] Li X. Zhu H. Geisen S. Bellard C. Hu F. Li H.. (2020b). Agriculture erases climate constraints on soil nematode communities across large spatial scales. Global Change Biology. 26, 919–930. 10.1111/gcb.14821 31479174

[B31] Lin K. Xiaoqing X. U. Jin X. Shao Z. Xiang Y. (2005). Eco-toxicological effects of selenium and its critical value on *Oryza sativa*. Chin. J. Appl. Ecol. 16, 678–682. 16011166

[B32] Liu C. C. Liu Y. G. Guo K. Qiao X. G. Zhao H. W. Wang S. J.. (2018). Effects of nitrogen, phosphorus and potassium addition on the productivity of a karst grassland: plant functional group and community perspectives. Ecol. Eng. 117, 84–95. 10.1016/j.ecoleng.2018.04.008

[B33] Liu T. Whalen J. K. Ran W. Shen Q. Li H. (2016). Bottom-up control of fertilization on soil nematode communities differs between crop management regimes. Soil Biol. Biochem. 95, 198–201. 10.1016/j.soilbio.2016.01.005

[B34] Long Z. D. Xiang J. Q. Song J. P. Lu Y. P. Yin H. Q. Zhu Y. F.. (2020). Soil selenium concentration and residents daily dietary intake in a selenosis area: a preliminary study in Yutangba Village, Enshi City, China. Bull. Environ. Contamin. Toxicol. 105, 798–805. 10.1007/s00128-020-02983-x 32909074

[B35] Lv Q. Liang X. Nong K. Gong Z. Qin T. Qin X.. (2021). Advances in research on the toxicological effects of selenium. Bull. Environ. Contam. Toxicol. 106, 715–726. 10.1007/s00128-020-03094-3 33420800

[B36] Mojtaba S. Naser S. M. Amin A. (2015). Antifungal activity of selenium nanoparticles synthesized by *Bacillus species* Msh-1 against *Aspergillus fumigatus* and *Candida albicans*. Jundishapur J. Microbiol. 8:e26381. 10.5812/jjm.26381 26495111 PMC4609177

[B37] Mora M. L. Duran P. Acuna A. J. Cartes P. Demanet R. Gianfreda L. (2015). Improving selenium status in plant nutrition and quality. J. Soil Sci. Plant Nutrition 15, 486–503. 10.4067/S0718-95162015005000041

[B38] Moulick D. Ghosh D. Santra S. C. (2016). Evaluation of effectiveness of seed priming with selenium in rice during germination under arsenic stress. Plant Physiol. Biochem. 109, 571–578. 10.1016/j.plaphy.2016.11.004 27838598

[B39] Moulick D. Santra S. C. Ghosh D. (2018). Effect of selenium induced seed priming on arsenic accumulation in rice plant and subsequent transmission in human food chain. Ecotoxicol. Environ. Safety 152, 67–77. 10.1016/j.ecoenv.2018.01.037 29407784

[B40] Mu S. Yamaji N. Sasaki A. Luo L. Du B. B. Che J.. (2021). A transporter for delivering zinc to the developing tiller bud and panicle in rice. Plant J. 105, 786–799. 10.1111/tpj.15073 33169459

[B41] Neher D. A (2001). Role of nematodes in soil health and their use as indicators. Journal of Nematology. 33, 161–168. 19265875 PMC2620512

[B42] Okada H. Niwa S. Takemoto S. Komatsuzaki M. Hiroki M. (2011). How different or similar are nematode communities between a paddy and an upland rice field across a flooding–drainage cycle? Soil Biol. Biochem. 43, 2142–2151. 10.1016/j.soilbio.2011.06.018

[B43] Park B. Y. Lee J. K. Ro H. M. Kim Y. H. (2011). Effects of heavy metal contamination from an abandoned mine on tomato growth and root-knot nematode development. Plant Pathology J. 27, 266–271. 10.5423/PPJ.2011.27.3.266

[B44] Park B. Y. Lee J. K. Ro H. M. Kim Y. H. (2016). Short-term effects of low-level heavy metal contamination on soil health analyzed by nematode community structure. Plant Pathol. J. 32, 329–339. 10.5423/PPJ.OA.12.2015.0272 27493608 PMC4968643

[B45] Paz-Ferreiro J. Fu S. L. (2016). Biological indices for soil quality evaluation: perspectives and limitations. Land Degradation Dev. 27, 14–25. 10.1002/ldr.2262

[B46] Prins C. N. Hantzis L. J. Valdez-Barillas J. R. Cappa J. J. Pilon-Smits E. (2019). Getting to the root of selenium hyperaccumulation-localization and speciation of root selenium and its effects on nematodes. Soil Syst. 3:47. 10.3390/soilsystems3030047

[B47] Rayman M. P (2000). The importance of selenium to human health. Lancet. 356, 233–241. 10.1016/S0140-6736(00)02490-9 10963212

[B48] Ros G. H. Van Rotterdam A. M. D. Bussink D. W. Bindraban P. S. (2016). Selenium fertilization strategies for bio-fortification of food: an agro-ecosystem approach. Plant Soil. 404, 99–112. 10.1007/s11104-016-2830-4

[B49] Sharma S. Bansal A. Dogra R. Dhillon S. K. Dhillon K. S. (2011). Effect of organic amendments on uptake of selenium and biochemical grain composition of wheat and rape grown on seleniferous soils in northwestern India. J. Plant Nutr. Soil Sci. 174, 269–275. 10.1002/jpln.200900265

[B50] Somogyi Z. Répási V. Tímár Á. Nagy P. Kiss I. Kád á,r I.. (2006). “Dose-dependent toxic effects of selenium on faunal elements of soil food web,” in Proceedings of the Trace Elements in the Food Chain (IMEC HAS, Budapest), 56–60.

[B51] US Environmental Protection Agency (2005). Guidance for Developing Ecological Soil Screening Levels. Available online at: https://www.epa.gov/sites/production/files/2015-09/documents/ecossl_guidance_chapters.pdf (accessed November 20, 2017).

[B52] Wang J. Wang Z. Mao H. Zhao H. Huang D. (2013a). Increasing Se concentration in maize grain with soil- or foliar-applied selenite on the Loess Plateau in China. Field Crops Res. 150, 83–90. 10.1016/j.fcr.2013.06.010

[B53] Wang Y. D. Wang X. Wong Y. S. (2013b). Generation of selenium-enriched rice with enhanced grain yield, selenium content and bioavailability through fertilization with selenite. Food Chem. 141, 2385–2393. 10.1016/j.foodchem.2013.05.095 23870972

[B54] Winkel L. H. Vriens B. Jones G. D. Schneider L. S. Pilon-Smits E. Bañuelos G. S. (2015). Selenium cycling across soil-plant-atmosphere interfaces: a critical review. Nutrients 7, 4199–4239. 10.3390/nu7064199 26035246 PMC4488781

[B55] Wu Z. L. Banuelos G. S. Lin Z. Q. Liu Y. Yuan L. X. Yin X. B.. (2015). Biofortification and phytoremediation of selenium in China. Front. Plant Sci. 6:136. 10.3389/fpls.2015.00136 25852703 PMC4367174

[B56] Xiao K. Song M. Liu J. Chen H. Li D. Wang K. (2018). Differences in the bioaccumulation of selenium by two earthworm species (*Pheretima guillemi* and *Eisenia fetida*). Chemosphere 202, 560–566. 10.1016/j.chemosphere.2018.03.094 29597172

[B57] Xu Q. Y. Shao X. Q. Shi Y. J. Qian L. Zhou X. Qing W. Y.. (2022). Is selenium beneficial or detrimental to earthworm? growth and metabolism responses of *Eisenia Fetida* to Na_2_SeO_3_ exposure. Sci. Total Environ. 807:150770. 10.1016/j.scitotenv.2021.150770 34624283

[B58] Yang D. D. Hu C. X. Wang X. Shi G. Y. Li Y. F. Fei Y. C.. (2021). Microbes: a potential tool for selenium biofortification. Metallomics. 13:mfab054. 10.1093/mtomcs/mfab054 34477877

[B59] Yang F. Chen L. Hu Q. Pan G. (2003). Effect of the application of selenium on selenium content of soybean and its products. Biol. Trace Element Res. 93, 249–256. 10.1385/BTER:93:1-3:249 12835506

[B60] Yeates G. W (2003). Nematodes as soil indicators: functional and biodiversity aspects. Biol. Fertility Soils 37, 199–210. 10.1007/s00374-003-0586-5

[B61] Yeates G. W. Bongers T. De Goede R. G. Freckman D. W. Georgieva S. S. (1993). Feeding habits in soil nematode families and genera—an outline for soil ecologists. J. Nematol. 25, 315–331. 19279775 PMC2619405

[B62] Zhang X. K. Li Q. Wang S. B. Jiang Y. Liang W. (2006). Effect of zinc addition to soil on nematode community structure. Bull. Environ. Contamin. Toxicol. 76, 589–594. 10.1007/s00128-006-0960-8 16688539

[B63] Zhao C. Ren J. Xue C. Lin E. (2005). Study on the relationship between soil selenium and plant selenium uptake. Plant Soil. 277, 197–206. 10.1007/s11104-005-7011-9

[B64] Zhao J. Neher D. A. (2013). Soil nematode genera that predict specific types of disturbance. Appl. Soil Ecol. 64, 135–141. 10.1016/j.apsoil.2012.11.008

